# An introduction to the ISRII conference, Limerick, Ireland, June 2–5, 2024

**DOI:** 10.1016/j.invent.2024.100727

**Published:** 2024-02-13

**Authors:** Nick Titov

**Affiliations:** Macquarie University, North Ryde, Sydney, NSW 2109, Australia

The International Society for Research on Internet Interventions (ISRII) was founded in 2004 to support the growing community of researchers engaged in studying how the internet could be used to improve behavioral and mental health. Today ISRII aims to foster excellence in the development and testing of a broad range of digital technologies (including mobile devices, digital gaming, virtual reality, remote sensing, and robotics, among others) for the promotion, prevention, treatment, and maintenance of the health and wellbeing of individuals.

Based on the number of trials, published manuscripts, and presentations, ISRII has been enormously successful in helping develop and demonstrate how the internet and related digital technologies can be used to deliver a broad range of educational, social and clinical benefits to people around the world.

This year the ISRII conference (ISRII12) will be held at the University of Limerick, Ireland. The theme of ISRII12, *Twenty Years of ISRII: Reflection, Celebration, and the Future*, acknowledges and celebrates the enormous achievements to date and potential opportunities in the future.

The Conference Chair, Professor Pepijn Van de Ven, warmly welcomes you to register for this wonderful conference as a presenter and/or attendee.

Prior to the Conference start, there are four outstanding workshops available. The three-day Conference will be filled with speakers from around the world sharing their ideas, research outcomes and questions in a collegial and comfortable environment nestled in the heart of the beautiful campus of the University of Limerick.

Highlights of ISRII12 will include:

• Keynotes
  o Day 1: Co-presented by Professors Lee Ritterband, Gerhard Andersson, Heleen Riper, and Helen Christensen. This Keynote, titled, *In the Beginning*, will describe the aspirations, visions and context for ISRII.
  o Day 2: Presented by Professor David Mohr. This Keynote, titled *State of the Art*, will synthesise what researchers have found across the digital health space.
  o Day 3: Co-presented by Dr. Sarah Vigerland, Dr. Alexis Whitton, and Associate Professor Charles Jonassaint. This Keynote, titled, *ISRII in 2044*, will introduce the presenters' individual work and their vision for ISRII and for our field in 20 years time.
• Early Career Researcher (ECR) meetings. Events are being planned to support ECRs to network, meet senior researchers, and enjoy!
• Workshops. A range of rich, engaging and practical workshops are planned to support the learning of knowledge and skills and their application.
• Awards. ISRII members will be recognised through an awards' ceremony, which ISRII can nominate people for via the ISRII.org website.
• Social Events. The Conference will host several relaxed social events to foster a collegial and unforgettable experience, including the Conference Dinner which will include traditional Irish singing and dancing.

Please visit isrii.org for more information and to register. Email info@isrii.org with any questions. We look forward to welcoming you to ISRII12.

Nick Titov and Louise Thorton, on behalf of the ISRII12 Conference Committee.

## Declaration of competing interest

The authors declare that they have no known competing financial interests or personal relationships that could have appeared to influence the work reported in this paper.

